# Few amino acid positions in *rpoB *are associated with most of the rifampin resistance in *Mycobacterium tuberculosis*

**DOI:** 10.1186/1471-2105-5-137

**Published:** 2004-09-28

**Authors:** Michael P Cummings, Mark R Segal

**Affiliations:** 1 Center for Bioinformatics and Computational Biology, University of Maryland, College Park, MD 20742-3360, USA; 2 Department of Epidemiology and Biostatistics, University of California, San Francisco, CA 94143-0560, USA

## Abstract

**Background:**

Mutations in *rpoB*, the gene encoding the *β *subunit of DNA-dependent RNA polymerase, are associated with rifampin resistance in *Mycobacterium tuberculosis*. Several studies have been conducted where minimum inhibitory concentration (MIC, which is defined as the minimum concentration of the antibiotic in a given culture medium below which bacterial growth is not inhibited) of rifampin has been measured and partial DNA sequences have been determined for *rpoB *in different isolates of *M. tuberculosis*. However, no model has been constructed to predict rifampin resistance based on sequence information alone. Such a model might provide the basis for quantifying rifampin resistance status based exclusively on DNA sequence data and thus eliminate the requirements for time consuming culturing and antibiotic testing of clinical isolates.

**Results:**

Sequence data for amino acid positions 511–533 of *rpoB *and associated MIC of rifampin for different isolates of *M. tuberculosis *were taken from studies examining rifampin resistance in clinical samples from New York City and throughout Japan. We used tree-based statistical methods and random forests to generate models of the relationships between *rpoB *amino acid sequence and rifampin resistance. The proportion of variance explained by a relatively simple tree-based cross-validated regression model involving two amino acid positions (526 and 531) is 0.679. The first partition in the data, based on position 531, results in groups that differ one hundredfold in mean MIC (1.596 *μg/ml *and 159.676 *μg/ml*). The subsequent partition based on position 526, the most variable in this region, results in a > 354-fold difference in MIC. When considered as a classification problem (susceptible or resistant), a cross-validated tree-based model correctly classified most (0.884) of the observations and was very similar to the regression model. Random forest analysis of the MIC data as a continuous variable, a regression problem, produced a model that explained 0.861 of the variance. The random forest analysis of the MIC data as discrete classes produced a model that correctly classified 0.942 of the observations with sensitivity of 0.958 and specificity of 0.885.

**Conclusions:**

Highly accurate regression and classification models of rifampin resistance can be made based on this short sequence region. Models may be better with improved (and consistent) measurements of MIC and more sequence data.

## Background

Rifampin, one of the principal drugs used in tuberculosis treatment, is a semi-synthetic antibiotic that inhibits transcription by preventing RNA synthesis. Isolates of *Mycobacterium tuberculosis *resistant to rifampin occur at low to moderate frequencies in many regions of the world [1]. Mutations in *rpoB*, the gene encoding the *β *subunit of DNA-dependent RNA polymerase, are associated with rifampin resistance. In the laboratory, drug resistance is quantified in terms of minimum inhibitory concentration (MIC), which is defined as the minimum concentration of the antibiotic in a given culture medium below which bacterial growth is not inhibited.

Several studies have been conducted where MIC of rifampin has been measured and partial DNA sequences have been determined for *rpoB *in different isolates of *M. tuberculosis *[2-6]. However, no model has been constructed to predict rifampin resistance based on sequence information alone. Such a model might provide the basis for quantifying rifampin resistance status based exclusively on DNA sequence data and thus eliminate the requirements for time consuming culturing and antibiotic testing of clinical isolates. Tree-based statistical methods (see Methods) have generated very accurate models relating amino acid sequence of short (8-mer) peptides to their binding by major histocompatibility complex (MHC) class I molecules with higher accuracy than artificial neural networks [7]. Both tree-based models and aggregation of such models through random forests (see Methods) have proven to be quite successful in other problems involving sequence data as covariates such as HIV-1 replication capacity [8] and cytidine to uridine RNA editing in plant mitochondria [9]. The success of tree-based statistical models and random forests in these problems involving covariates derived from sequence data motivated our application of these models to the problem of rifampin resistance in *M. tuberculosis*.

The response variable is a set of continuously distributed values for MIC, which makes the problem one of regression. These data are used to answer the following questions: What proportion of the variance in MIC is attributable to sequence differences in positions 511–533 of the *β *subunit of RNA polymerase of *M. tuberculosis*? What particular positions, and what distribution of amino acids at those positions, are associated with most of the variance in MIC? Alternatively, the response variable could be cast in discrete terms: resistant or susceptible. This is possible by assuming a threshold value for MIC above which an isolate is considered resistant to rifampin. Among the specific questions we can answer with such a model are the following: What particular positions, and what distribution of amino acids at those positions, allow for distinguishing rifampin-susceptible and rifampin-resistant isolates of *M. tuberculosis*? What is the misclassification error rate associated with susceptibility prediction for these data? We address these questions and evaluate the ability to predict MIC from protein sequence data (inferred from DNA sequence data) using tree-based regression and classification methods. We find that these methods generate highly accurate models of rifampin resistance.

## Results

The data set used in the study consists of 173 observations with 60 distinct genotype-phenotype combinations (Table 1). The most frequent combination has 47 occurrences, and there are 40 unique (singleton) combinations. MIC for rifampin varies from 0.0625 *μg/ml *to > 512 *μg/ml*. The 173 sequences are distributed among 24 genotypes, 11 of which occur uniquely in the data set. The plurality genotype is represented by 69 samples; 98 samples differ from the plurality by one amino acid; and the remaining 6 samples differ from the plurality by two amino acids. Some genotypes defined by the partial sequence of *rpoB *are associated with several different phenotypes (MIC values). Also, some genotypic states are associated with large effects, while some have little or no effect on MIC phenotype. Finally, some changes in MIC are not associated with changes in the sequence region examined. These genotype data are short (69 bp) partial sequences of a single gene, and thus they may not contain all phenotypically relevant genetic information. Indeed, there is evidence that amino acid changes outside of the examined region are associated with changes in MIC for rifampin [3]. Additionally, the sample size is small (also typical of most genotype-phenotype datasets), which will decrease power. Nonetheless these data are typical of studies surveying the genetic variation associated with antibiotic resistance and of genotype-phenotype data in general. Thus they make an appropriate subject of investigation.

### Regression tree analysis

**Figure 1:**
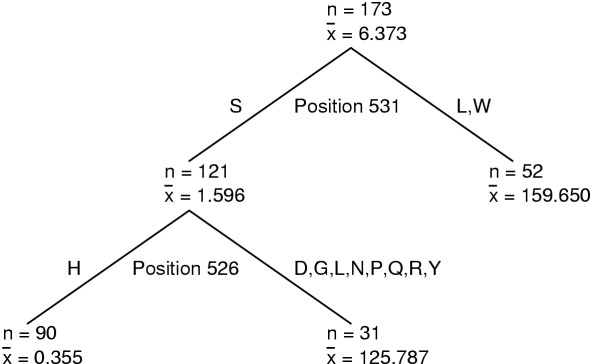
**Cross-validated pruned regression tree for minimum inhibitory concentration (MIC) of rifampin (*μg/ml*) based on amino acid sequence data from positions 511–533 of the *β *subunit of RNA polymerase of *Mycobacterium tuberculosis*. **The number of observations (n) and the mean MIC values across observations (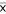) are given for each node. Split definitions are depicted with amino acid positions represented by numerals and amino acids represented by single letter code adjacent branches.

The regression tree for the relationship of *rpoB *amino acid sequence and MIC has two splits defining three terminal nodes (Fig. 1). At each node in the tree, the MIC prediction given (*μg/ml*) is the mean of all isolates at that node. The first split of the topmost node (root node) consists of the entire sample and is based on the amino acid at position 531, with those sequences having serine (S) going to the left child node, and those having leucine (L) or tryptophan (W) going to the right child node. The best split for each node is that which gives the largest decrease in the error. Here error is measured as the deviance, which for a continuous variable is a constant multiple of the residual sums-of-squares. Reported values were determined using 10-fold cross-validation. Moving down the tree the error decreases, as the sum of the deviance for each pair of child nodes is less than the deviance of the parent node. Given the hierarchical nature of trees and the criterion used to choose splits, the first split, that based on position 531, explains the highest proportion of the overall phenotypic variance. This bisection of the data results in groups that differ one hundredfold in mean MIC (1.596 *μg/ml *and 159.676 *μg/ml*). The subsequent partition based on position 526, the most variable in this region, results in a > 354-fold difference in MIC. The proportion of the variance in MIC explained across all splits involving the two amino acid positions (526 and 531) is 0.679. All proportions of variances explained by the model as reported here are those estimated through cross-validation and are not based on re-substitution, and thus represent appropriately conservative estimates.

### Classification tree analysis

From a clinical perspective it may be most relevant to consider the level of drug resistance as a two-state categorical variable (susceptible or resistant) rather than as a continuously distributed variable. In clinical practice, if an isolate of *M. tuberculosis *is determined to be rifampin resistant then rifampin is replaced with another antibiotic. Although blood serum concentration of rifampin reaches levels of 6 – 7 *μg/ml *about 1.5 – 2 hours after ingestion [10], a clinically relevant MIC value for dichotomizing the MIC values would be lower than this peak. We conservatively define MIC values ≤ 1 *μg/ml *as susceptible and values > 1 *μg/ml *as resistant, a definition consistent with conventional standards [11]. With this dichotomization we can explore the use of tree-based statistical classification to predict rifampin resistance in a way that is more relevant to clinical practice.

**Figure 2:**
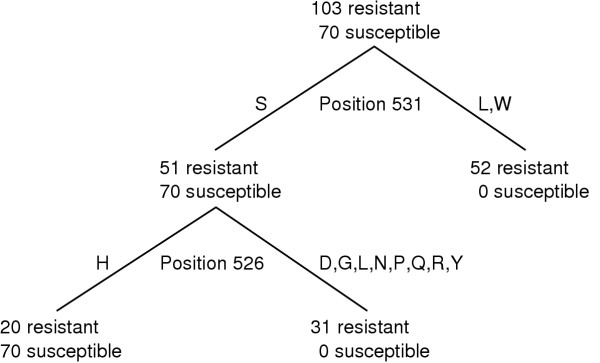
**Cross-validated pruned classification tree for rifampin susceptibility and resistance based on amino acid sequence data from positions 511–533 of the *β *subunit of RNA polymerase of *Mycobacterium tuberculosis*. **The numbers of observations in each class, susceptible or resistant, are given for each node.

The predictor variables are again the unordered categorical designations of amino acids at polymorphic positions. The classification tree for these data (Fig. 2) has two splits based on two of the 11 variable amino acid positions. At each node in the tree, the prediction of rifampin susceptibility status (susceptible or resistant) is given for all isolates at that node. The first split is based on position 531; those isolates with serine (S) are predicted to be susceptible, and those with leucine (L) or tryptophan (W) are predicted to be resistant. The class counts for the full data set are given at each node. For example, the root node (top most node in the figure) contains all 173 cases of which 103 are resistant are resistant to rifampin, and the remaining 70 isolates are susceptible to rifampin. The proportion of correctly classified observations across all splits as determined by re-substitution of the observations on the cross-validation pruned subtree is 0.884. Comparing this tree to the pruned regression subtree (Fig. 1) reveals that the two split definitions in each tree are identical. Both the regression and classification tree models are significant (*P *< 0.0001) based on permutation tests.

### Random forest analyses

The random forest analysis, which aggregates results over many tree models, each constructed on subsamples of the data, produced markedly better models as compared to the single tree-based models. The random forest analysis of the MIC data as a continuous variable, a regression problem, produced a model that explained 0.861 of the variance. The random forest analysis of the MIC data as discrete classes (susceptible and resistant), a classification problem, produced a model that correctly classified 0.942 of the observations with corresponding sensitivity of 0.958 and specificity of 0.885.

**Figure 3:**
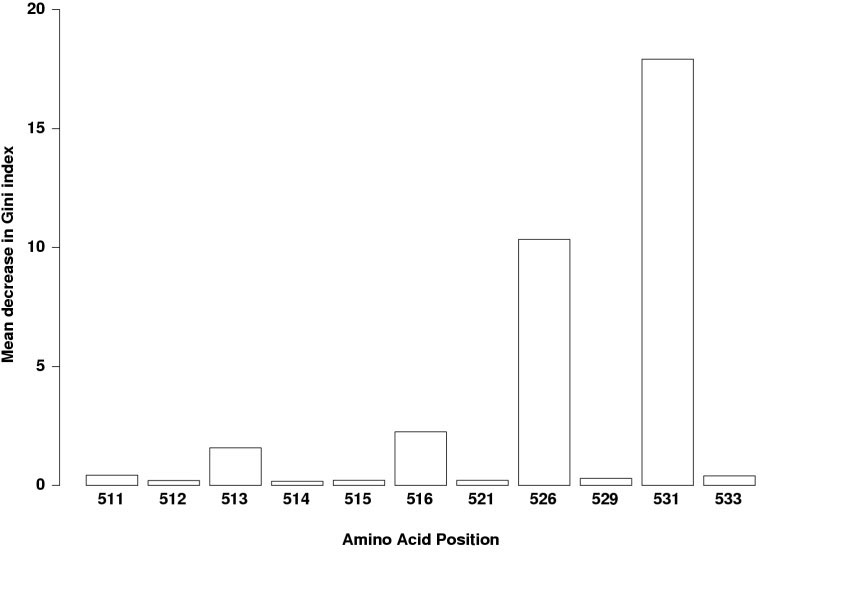
**Variable importance plot from random forest classification analysis. **The plot includes all polymorphic positions in the region examined, and shows the importance of each position as the decrease in the Gini index (a measure of impurity) induced by splitting the data on that position averaged over all trees (higher values are more important). The plot for regression analysis is very similar (not shown).

Although both the regression and classification random forest results are markedly better than the single tree-based models, they do lack the ease of interpretation of a tree model. However, variable importance can be assessed in random forests by measuring the increase in group purity based individual models containing the variable. As might be expected, the results for both regression and classification are similar and identify the same amino acid positions as being most important in determining response to rifampin as did the single tree models: primarily 531 and 526, and much less so for 513 and 516 (Figure 3).

## Discussion

Analysis of genotype and phenotype data poses several significant challenges. Data characteristics such as mixture of variable types, high dimensionality, interactions between variables, and preponderance of unordered categorical variables render many candidate analytical methods inappropriate or ineffective. Tree-based statistical models adeptly deal with these all these challenges and do so in a way that produces readily interpretable results.

Through the analyses described above, we have learned several things that were not previously apparent. We have distinguished phenotypically relevant from phenotypically irrelevant changes in genotype by establishing the relative importance of the polymorphic sequence positions, and amino acids at those positions, as they affect susceptibility to rifampin. For example, although they are polymorphic, changes at positions 511, 512, 515, 521 and 529 did not significantly affect MIC for rifampin. The hierarchical importance of changes, and their contextual/conditional relationships, are depicted in the resulting tree diagrams in a readily interpretable manner. Inherent in the tree structure is the fact that earlier splits explain more variation in phenotype then subsequent splits. For example, the first split, at position 531, explains more variation then does the split based on position 526.

The models can be used to predict MIC for rifampin where genotype is known, as well as provide the basis for hypothesis testing involving future empirical work. Furthermore models can be refined to yield improved predictions by incorporating additional data as they become available. Improved models may be possible with additional data: full length sequence of *rpoB *may include sequence features that are responsible for some variation in MIC values for rifampin, and sequence data from additional strains might lead to even more general models.

As demonstrated above, the relationship of genotype to phenotype can be quantified using tree-based statistical models and aggregations thereof. Our approach has been to use types of models in the analysis of genotype-phenotype relationships because they offer distinct advantages compared to other methods and allow for rigorous and ready interpretation of results. Tree-based and random forest analyses are readily applicable to other forms of genotypic information including data that take the general form of visualized fragments (bands on gels) such as microsatellites, restriction fragment length polymorphisms (RFLPs), amplified fragment length polymorphisms (AFLPs), and similar data. Tree-based and random forest analyses can also be applied directly to DNA sequence data including single nucleotide polymorphisms (SNPs). In general, tree-based statistical and random forest models are applicable to all cases where the goal is to examine the relationship between genotype and phenotype.

## Conclusions

Relatively simple models provided accurate predictions of rifampin resistance in *M. tuberculosis*. These models demonstrated that only a few variable positions in the *β *subunit of DNA-dependent RNA polymerase were responsible for most of the variation in rifampin resistance. Such models might provide the basis for quantifying rifampin resistance status based exclusively on DNA sequence data and thus eliminate the requirements for time consuming culturing and antibiotic testing of clinical isolates. More generally, the results of this study demonstrate the usefulness of tree-based statistical models and random forests in genetic analysis.

## Methods

### Data sources

Sequence data for amino acid positions 511–533 of *rpoB *and associated MIC of rifampin for different isolates of *M. tuberculosis *were taken from studies examining rifampin resistance in clinical samples from New York City and throughout Japan [2-4]. Minimum inhibitory concentration (MIC) is defined as the minimum concentration of the antibiotic in a given culture medium below which bacterial growth is not inhibited.

### Variables

The predictor variables are unordered categorical designations of amino acid at polymorphic positions, and the response variable are continuous values for MIC represented by their *log*_2 _transforms. Values given in the original sources as <*x *were set to log_2_(*x *- 0.5), and those values given as >*x *were set to log_2_(*x *+ 0.5). The MIC values are converted back to *μg/ml *in figures to be consistent with Table 1 and to facilitate interpretation.

### Tree-based statistical analyses

Analysis of the relationships between *rpoB *amino acid sequence and rifampin susceptibility was done through the use of tree-based statistical models [12], also known as classification and regression trees (CART) [13]. Analyses were done with rpart (recursive partitioning) [14] using the rpart library [15] for the open source statistical package R [16]. Tree-based models operate by recursively partitioning a data set in two (binary split) based on the value of a single predictor variable to best achieve homogeneous collections of a nominal or ordinal response variable (classification) or to best separate low and high values of a continuous response variable (regression). The split definition can be considered as a question, which has the following general form: Is the observation **x**_*i *_∈ *A*? Here *A *is a region of the variable space. Thus answering the question for all observations produces two groups of observations; those for which the answer is yes (those in region *A*) and those for which the answer is no (**x**_*i *_∉ *A*, those in the complement of *A*). The specific criteria for choosing among the possible partitions (questions) is based on the change in deviance, which for regression problems is equivalent to least squares. Subsequent binary partitioning continues until stopping criteria (variously defined) are met. The result is a classification or regression tree: a hierarchical series of data bifurcations, which depicts the partition definitions and describes the resulting data subsets defined by each partition.

For unordered categorical covariates, such as amino acid designation, the search through possible splits is exhaustive. For each variable amino acid position there are 2^*n*-1 ^-1 possible partitions, where *n *is the number of different amino acids observed. For example, in the case of amino acid position 526 of *rpoB *analyzed here there are 9 observed amino acids resulting in 255 possible partitions to be evaluated. The preferred way to construct an appropriately sized tree is to first build a large tree and subsequently prune it [12,13]. Pruning is the process of removing branches from a tree to produce a subtree. To objectively choose the appropriate size for a pruned tree, it is useful to employ the concept of *cost-complexity *[13]. Embodied within the cost-complexity measure is a reward for tree (model) fit and a penalty for tree size (number of parameters). A tree can be pruned by using the cost-complexity measure to identify subtrees to be eliminated. A more formal definition and discussion of cost-complexity is given elsewhere [13].

Performance of tree-based models can be assessed in a number of ways depending on the goals of the analysis. One way is to evaluate the fit of the data used to generate the model, which is known as the re-substitution error. The use of re-substitution error may be justified when the principal goal of the analysis is to explain the observations in hand. However, the re-substitution error provides an underestimate of the error if the goal is to produce a model for future prediction. Another scheme to assess performance is to partition observations into a subset for model building, the training set, and a subset to evaluate the model, the test set. To remove biases this general scheme can be expanded in the form of cross-validation. Typically 10-fold cross-validation is used, where the data are randomly divided into 10 equal or near equal portions. Nine of these portions are used to generate the model and the remaining portion, the test set, is used to evaluate the model. This step is repeated until all test sets have been used in model evaluation.

We assessed the significance of our tree-based statistical models through permutation where the predictor variables are randomized with respect to the response variable [17]. The frequency of observing a result value equal to or better than the observed value in 1 × 10^4 ^permutations is the estimate of the probability associated with the observed result.

### Random forest analyses

In a series of recent papers [18-21], Breiman has demonstrated that consequential gains in classification or prediction accuracy can be achieved by using an ensemble of trees, where each tree in the ensemble is grown in accordance with the realization of a random vector. Final predictions are obtained by aggregating (voting) over the ensemble, typically using equal weights. Bagging [18] represents an early example whereby each tree is constructed from a bootstrap [22] sample drawn with replacement from the training data. The simple mechanism whereby bagging reduces prediction error for unstable predictors, such as trees, is well understood in terms of variance reduction resulting from averaging [18,23]. Such variance gains can be enhanced by reducing the correlation between the quantities being averaged. It is this principle that motivates random forests.

Random forests seek to effect such correlation reduction by a further injection of randomness. Instead of determining the optimal split of a given node of a (constituent) tree by evaluating all allowable splits on all covariates, as is done with single tree methods or bagging, a subset of the covariates drawn at random is employed. Breiman [20,21] argues that random forests (a) enjoy exceptional prediction accuracy, (b) that this accuracy is attained for a wide range of settings of the single tuning parameter employed, and (c) that over-fitting does not arise due to the independent generation of ensemble members.

Here, our random forests comprised 1 × 10^4 ^individual trees constructed by sub-sampling eight predictor variables (regression) or two predictor variables (classification) at each node. Variable importance was assessed by measuring the increase in group purity when partitioning data based on a variable. We used the R package randomForest [24].

## Authors' contributions

MPC conceived of the study, and participated in its coordination. Both authors participated in study design, carried out the statistical analyses, wrote and approved the final manuscript.

## Figures and Tables

**Table 1:** Minimum inhibitory concentration (MIC) of rifampin and associated variable amino acids in positions 511–533 of the *β *subunit of RNA polymerase of *Mycobacterium tuberculosis *[2–4].

MIC (*μg/ml*)	No. of Isolates	No. of Isolates with Differences	Amino Acid Differences from Consensus (No. of Isolates)
0.0625	48	1	515:V(1)
0.125	2	0	
0.25	2	0	
< 0.39	13	2	521:P(1), 533:P(1)
0.5	2	1	533:P(1)
1	3	1	533:P(1)
2	2	1	511:R and 512:T(1)
4	2	1	516:V(1)
8	6	5	516:V(1), 526:G(1), 526:L(2), 526:Q and 533:P(1)
12.5	3	3	514:L and 516:V(1), 533:P(2)
16	3	3	526:L(1), 526:N(1), 529:K(1)
32	1	0	
> 32	15	15	511:R and 516:Y(1), 513:K(1), 513:L(1), 526:D(2), 526:Y(4), 531:L(4), 531:W(1), 533:P(1)
50	1	1	531:L(1)
64	7	7	531:L(7)
100	1	1	531:L(1)
128	19	19	513:L(1), 516:Y(1), 526Y(1), 531L(16)
200	1	1	526:D(1)
> 200	7	7	513:L(1), 516:A and 526:D(1), 526:P(1), 526:Y(2), 531:L(2)
256	13	13	513:K(1), 516:Y and 526:N(1), 526:P(1), 531:L(10)
512	18	18	526:P(4), 526:R(2), 526:Y(3), 531:L(7), 531:W(2)
> 512	4	4	516:Y(1), 526:D(1), 526:P(1), 531:W(1)
